# CancerSubtypeXplore: A Modular Platform for Multiomics Cancer Subtype Prediction and Biomarker Consensus Discovery

**DOI:** 10.34133/csbj.0030

**Published:** 2026-04-13

**Authors:** Dingnan Jin, Bian Bian, Yutaka Saito

**Affiliations:** ^1^ Department of Computational Biology and Medical Science, Graduate School of Frontier Sciences, The University of Tokyo, Kashiwa, Chiba 277-0882, Japan.; ^2^ Department of Data Science, School of Frontier Engineering, Kitasato University, Sagamihara, Kanagawa 252-0373, Japan.; ^3^ Artificial Intelligence Research Center, National Institute of Advanced Industrial Science and Technology (AIST), Tokyo 135-0064, Japan.

## Abstract

Identifying reproducible biomarkers and cancer subtypes from large-scale multiomics data remains a challenging task, as current computational frameworks often require coding expertise and lack standardized datasets for model benchmarking and exploration. To address these limitations, we developed CancerSubtypeXplore, a modular and user-friendly platform for multiomics cancer subtype prediction and biomarker discovery. The system integrates 4 main components: (a) a dataset module providing standardized and curated multiomics datasets from 17 The Cancer Genome Atlas cancer types, including mRNA, DNA methylation, and microRNA profiles; (b) a machine learning module for automated benchmarking using classical algorithms such as support vector machines and random forests; (c) design your deep learning model module that allows users to design and train customized neural network architectures without coding; and (d) a biomarker analysis module that extracts prediction-contributed features as biomarkers from each trained model, computes their intersections, and ranks them by frequency to identify robust cross-model or cross-cancer biomarkers. Benchmark experiments demonstrate consistent subtype prediction accuracy across multiple cancer types and reveal overlapping biomarkers that may serve as potential pan-cancer signatures. CancerSubtypeXplore provides a transparent, reproducible, and extensible environment for biomedical researchers to explore multiomics datasets, evaluate diverse models, and identify robust biomarkers.

## Graphical Abstract

**Figure:**
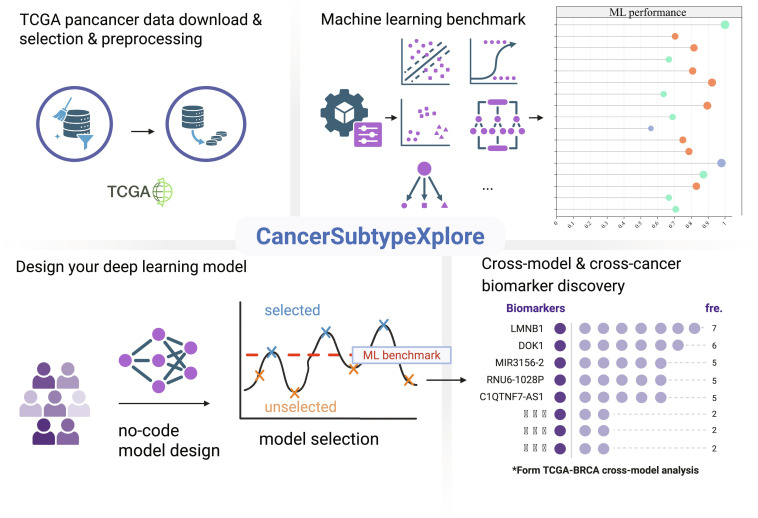

## Introduction

The Cancer Genome Atlas (TCGA) has become a cornerstone resource in cancer research, having molecularly characterized over 11,000 specimens across more than 30 cancer types, spanning genomic, transcriptomic, epigenomic, and proteomic data layers [1,2]. Multiomics data have enabled many studies to classify tumors into distinct molecular subtypes, discover prognostic biomarkers, and reveal regulatory mechanisms, showing clear advantages over single-omics analyses in robustness, biological insight, and clinical relevance [3–5].

In the context of cancer subtype delineation, multiple computational frameworks have been proposed to fuse heterogeneous molecular data types, such as mRNA expression, DNA methylation, microRNA (miRNA), copy number changes, and protein measurements. For example, the DeepProg framework combined transcriptomic, methylation, and miRNA data across 32 TCGA cancers, applying an ensemble of deep learning and classical methods to stratify prognostic groups and derive subtype-specific signatures [6]. The DeepMoIC model further leveraged graph-based deep learning to capture cross-omic dependencies, integrating RNA sequencing and copy number variation data from TCGA pan-cancer datasets to enhance subtype classification [7]. Subtype-GAN used a generative adversarial network architecture to integrate multiple molecular modalities across 10 TCGA cancers for subtype identification [8]. In hepatocellular carcinoma, a deep-learning-based integration of multiomics data achieved more accurate survival prediction than single-omics models [9]. The landscape review “deep-learning-driven multiomics analysis” further highlights the growing role of neural network architectures in tasks such as subtype classification, biomarker prioritization, and prediction of clinical outcomes [10].

Despite these advances, several challenges remain. First, data preprocessing and feature alignment often differ across studies, making model comparison and reproducibility difficult. Second, almost all of the existing methods fix architecture parameters, providing limited flexibility for users to tailor models to their own data characteristics or experiment with alternative designs. Third, while deep learning models often deliver strong predictive performance, their internal feature selection or ranking mechanisms are rarely exposed, making it harder to extract stable biomarkers across models. Furthermore, the top *k* features identified by different models often differ, making it difficult to reach a consistent biomarker consensus and to conduct biological validation. Several integrative frameworks have attempted to address parts of the multiomics analysis workflow, yet most remain limited in either flexibility or accessibility. For example, MOVICS provides an R-based toolkit for cancer subtyping and visualization through consensus clustering and survival analysis, but it requires scripting skills and focuses mainly on unsupervised learning [11]. DeepMoIC uses graph convolutional networks to model cross-omics relationships and improve subtype prediction yet lacks an interactive interface for model comparison [7]. DeepMOIS-MC extends deep-learning-based integration to prognostic prediction, but its fixed architecture and parameters restrict user customization [12]. Building on these efforts, recent benchmarking projects such as the MLOmics suite have begun to offer standardized datasets and baseline models for cancer subtyping and classification using TCGA data [13]. However, there is still no fully integrated and modular platform that allows users to move seamlessly from standardized multiomics data to model comparison and biomarker extraction in a no-code or low-code environment.

**Fig. 1.**
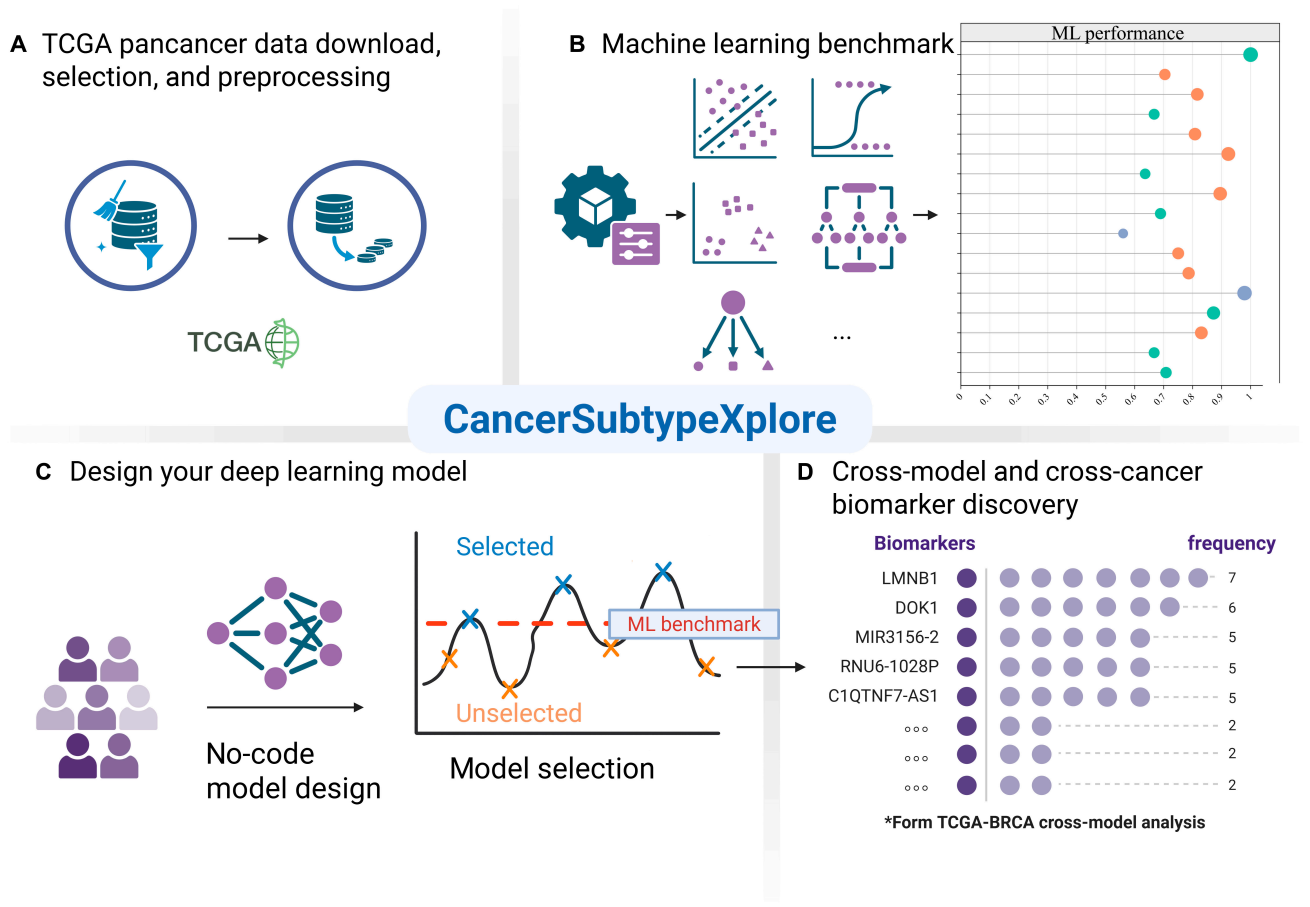
Overview of the CancerSubtypeXplore workflow. (A) Dataset preprocessing. Curated TCGA multiomics datasets (mRNA, DNA methylation, and microRNA [miRNA]) are presented after prior download, filtering, and normalization, ensuring consistent feature dimensions across datasets. (B) Machine learning benchmark. Classical algorithms (e.g., logistic regression, support vector machine [SVM], and random forest [RF]) are used to establish benchmark across cancers. (C) Deep learning model design. Users can interactively design and customize neural network architectures without coding, enabling intuitive exploration of model configurations that outperform machine learning benchmarks. (D) Cross-model and cross-cancer biomarker discovery. Contribution-based feature ranking combined with frequency aggregation identifies robust and cross-cancer biomarkers consistently highlighted across multiple deep learning models.

In response to this gap, we present CancerSubtypeXplore, a modular and extensible platform tailored for multiomics cancer subtype analysis and biomarkers discovery. Our architecture is divided into 4 modules (Fig. 1). The dataset module (Fig. 1A) curates and standardizes multiomics data from TCGA, harmonizing sample matching, normalization, and feature alignment across multiple cancer types. The machine learning (ML) module (Fig. 1B) allows users to benchmark classical classifiers (such as support vector machines [SVMs], random forests [RFs], and ridge classifier [Ridge]) on curated datasets without manual preprocessing. The deep learning module (Fig. 1C) empowers users to configure and train custom neural network architectures on the TCGA datasets, adjusting layer depth, hidden dimension, activation functions, and so on. Finally, the biomarker analysis module (Fig. 1D) extracts the top *k* ranked features from each trained model, measures their overlap across models or cancer types, and ranks them by occurrence frequency, thereby identifying robust biomarker candidates that are consistent across models and cancers.

We evaluate CancerSubtypeXplore across multiple TCGA cancer datasets to demonstrate its capacity for competitive subtype prediction, cross-model and cross-cancer biomarker discovery, and reproducibility. Through this work, we aim to provide a transparent, reproducible, and user-friendly platform that bridges the gap between advanced multiomics modeling methods and the practical needs of biomedical researchers, enabling them to identify stable biomarkers and generate mechanistic insights without requiring extensive coding expertise.

## Methods and Design

CancerSubtypeXplore was designed as a modular platform to support the major steps of practical multiomics subtype analysis within a unified workflow. Rather than introducing a single novel predictive algorithm, the platform integrates 4 functionally connected modules: dataset organization, classical ML benchmarking, configurable deep learning model design, and biomarker analysis. These modules were selected to provide complementary value. The dataset module standardizes heterogeneous multiomics inputs into a consistent format for downstream analysis; the ML module provides representative baseline comparisons across different learning paradigms; the deep learning module enables flexible no-code exploration of basic neural architectures and training settings; and the biomarker analysis module supports model-based feature prioritization for downstream biological interpretation. Together, these components are intended to improve usability, comparability, and exploratory robustness in subtype classification studies.

### Dataset

#### Data source and overall workflow

All datasets used in this study were obtained from TCGA project via the Genomic Data Commons (GDC) data portal [1,2]. TCGA provides large-scale, harmonized multiomics profiles across diverse cancer types, making it an ideal foundation for benchmarking ML and deep learning models in integrative cancer subtype analysis.

We limited our analysis to 17 TCGA datasets in which all 4 data modalities—mRNA, miRNA, DNA methylation array, and subtype labels—were simultaneously available under the harmonized GDC pipelines. TCGA profiled more than 30 cancer types with multiomics assays, but not every dataset has all modalities and curated subtypes concurrently for the same samples; using datasets lacking this complete overlap would preclude supervised subtype classification and bias biomarker discovery. By selecting projects with nonzero 4 modality intersections and subtypes, we ensure label consistency, platform comparability, and statistical adequacy for fair ML/deep learning benchmarking and feature attribution. In addition, for supplementary external validation, we included one independent breast cancer dataset, GEO-GSE96058_BRCA, which contains mRNA expression and subtype labels but does not include the miRNA and DNA methylation modalities used in the TCGA multiomics benchmark [14]. Therefore, this dataset was used only for external validation and enrichment analysis, rather than for the main multiomics benchmarking experiments.

Datasets were included only when reliable subtype labels were available and overlapping samples across all required modalities could be obtained after preprocessing. For supervised subtype classification, datasets lacking subtype annotations or having 50 or fewer intersected samples after preprocessing were excluded. The same threshold was applied across all cancer types in this study. Because subtype distributions varied across cancer types, the corresponding class composition for each included dataset was recorded to facilitate interpretation of benchmarking performance.

Two categories of datasets were constructed:

1. Ten independent TCGA datasets—kidney renal clear cell carcinoma (TCGA-KIRC), rectum adenocarcinoma (TCGA-READ), esophageal carcinoma (TCGA-ESCA), kidney chromophobe (TCGA-KICH), uterine carcinosarcoma (TCGA-UCS), kidney renal papillary cell carcinoma (TCGA-KIRP), adrenocortical carcinoma (TCGA-ACC), skin cutaneous melanoma (TCGA-SKCM), bladder urothelial carcinoma (TCGA-BLCA), and lung squamous cell carcinoma (TCGA-LUSC)—were used for specific cancer analyses.
2. Seven cross-cancer TCGA datasets—breast invasive carcinoma (TCGA-BRCA), colon adenocarcinoma (TCGA-COAD), head and neck squamous cell carcinoma (TCGA-HNSC), liver hepatocellular carcinoma (TCGA-LIHC), pheochromocytoma and paraganglioma (TCGA-PCPG), stomach adenocarcinoma (TCGA-STAD), and uterine corpus endometrial carcinoma (TCGA-UCEC)—were used for cross-cancer analyses.

Raw data were downloaded directly from the GDC using the TCGAbiolinks R package [15,16]. Each project included 3 modalities:

• mRNA expression (HTSeq counts or fragments per kilobase of transcript per million mapped reads)
• DNA methylation (Illumina Human Methylation 450K platform)
• miRNA expression (read counts)

Clinical subtype annotations were extracted from the pan-cancer atlas subtypes [17] resource whenever available. The standardized datasets, including training and evaluation sample sizes and class details, are summarized in Table S1.

#### Preprocessing for TCGA datasets

The 17 TCGA datasets were processed using a uniform R pipeline that automatically downloads, prepares, and filters samples across omics. For each project *c*, only primary tumor samples having all 3 omics data were retained:

$$S_{c}=S_{c}^{\left(\text{mRNA}\right)}\cap S_{c}^{\left(\text{Meth}\right)}\cap S_{c}^{\left(\text{miRNA}\right)}.$$ (1)

##### Zero and missing-value filtering

For each cancer dataset, we first examined every omics feature matrix to ensure data completeness and informativeness. Any feature column that consisted entirely of zero values or contained missing entries was excluded from further analysis. In this step, each omics type (including mRNA expression, DNA methylation, and miRNA expression) was treated separately, where each dataset comprised a defined number of patient samples and molecular features specific to that omics layer.

##### Transformation and standardization

To improve comparability and numerical stability across omics types, omics-specific value transformations were first applied during preprocessing. For mRNA data, transcripts per million (TPM) values were log-transformed as $\log_{2}\left(\mathrm{TPM}+1\right)$. DNA methylation *β* values were converted to *M* values $M=\log_{2}\beta /\left(1-\beta \right)$. miRNA read counts were log-scaled. After these modality-specific transformations, each feature was standardized as follows:

$${\tilde{x}}_{\mathrm{ij}}^{\left(k\right)}=\frac{x_{\mathrm{ij}}^{\left(k\right)}-\mu_{j}^{\left(k\right)}}{\sigma_{j}^{\left(k\right)}},$$ (2)

where $x_{\mathrm{ij}}^{\left(k\right)}$ denotes the value of feature j for sample i in omics type k, $\mu_{j}^{\left(k\right)}$ and $\sigma_{j}^{\left(k\right)}$ are the mean and standard deviation of feature j within omics k, and ${\tilde{x}}_{\mathrm{ij}}^{\left(k\right)}$ represents the standardized feature value used for subsequent analyses. These transformation and standardization steps serve different purposes and should not be interpreted as repeated application of the same normalization procedure.

The interface summarizes curated TCGA multiomics datasets, unified feature dimensions, and subtype annotations used for downstream ML and deep learning analyses.

##### Feature reduction

For the 10 independent TCGA datasets (TCGA-KIRC, TCGA-READ, TCGA-ESCA, TCGA-KICH, TCGA-UCS, TCGA-KIRP, TCGA-ACC, TCGA-SKCM, TCGA-BLCA, and TCGA-LUSC), variance-based filtering was applied until the target dimensionality per modality was reached:

$$p_{\text{mRNA}}^{*}=1,000,p_{\mathrm{DNA}\;\text{Meth}}^{*}=1,000,p_{\text{miRNA}}^{*}=500.$$ (3)

These thresholds balance computational feasibility and representativeness, following the preprocessing strategy of MOGONET [18].

##### Train/test partition

**Fig. 2.**
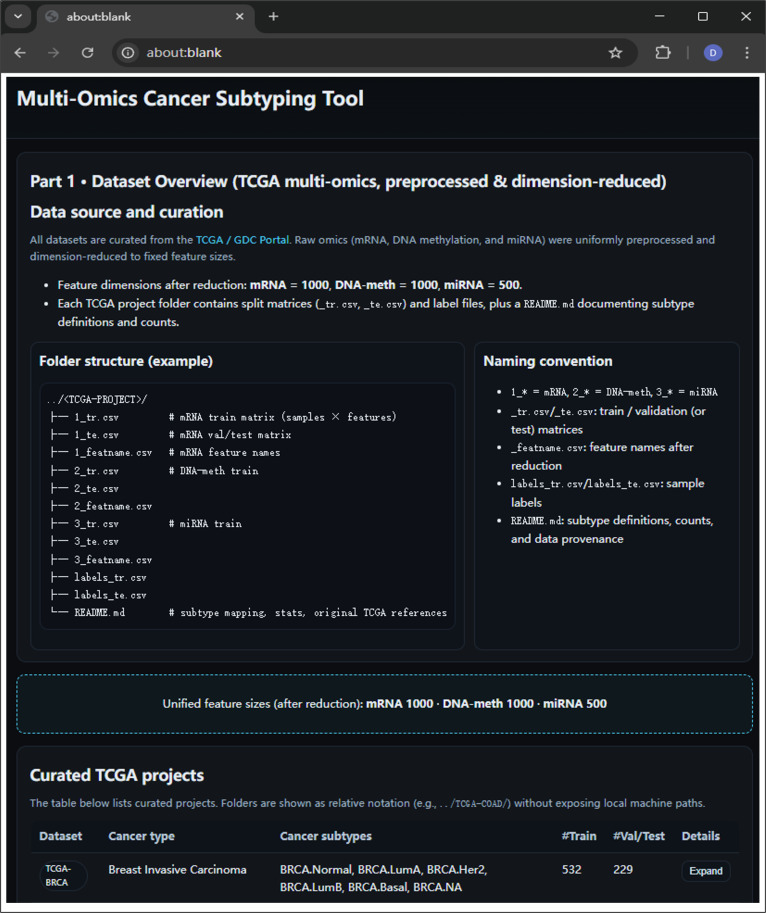
Preview of the CancerSubtypeXplore tool interface—part 1.

Each dataset was randomly split into training (70%) and testing (30%) subsets. Data were transposed to the sample × feature format and saved following the MOGONET file convention (Fig. 2):

• 1_* = mRNA, 2_* = DNA-meth, 3_* = miRNA
• _tr.csv/_te.csv: train/test matrices
• _featname.csv: feature names after reduction
• labels_tr.csv/labels_te.csv: sample labels
• README.md: subtype definitions, counts, and data provenance

#### Construction of the cross-cancer dataset

For the 7 cross-cancer TCGA datasets (TCGA-BRCA, TCGA-COAD, TCGA-HNSC, TCGA-LIHC, TCGA-PCPG, TCGA-STAD, and TCGA-UCEC), preprocessing followed the same general pipeline as that used for the independent datasets. For the independent datasets, dimensionality reduction was first performed separately for each omics modality, retaining 1,000 features for mRNA, 1,000 features for DNA methylation, and 500 features for miRNA.

To construct a unified cross-cancer dataset, an additional feature selection step was applied to retain only the features shared across all 7 cancer types. Specifically, the top variance features common to all 7 cancers were identified for each omics modality, so that every dataset contained an identical subset of cross-cancer features. Because biological heterogeneity across cancer types is substantial, the number of shared features remaining after this intersection step was limited on the order of a few hundred. Accordingly, the cross-cancer feature space was set to 200 mRNA features, 200 DNA methylation features, and 100 miRNA features.

For all datasets, the feature dimensions of 1,000 for mRNA, 1,000 for DNA methylation, and 500 for miRNA were set with reference to prior studies such as MOGONET. For the cross-cancer analysis, only features shared across all 7 cancer types were retained. After feature intersection, the numbers of shared features were just above 200 for mRNA and DNA methylation and 100 for miRNA; therefore, the cross-cancer feature space was defined as 200 mRNA features, 200 DNA methylation features, and 100 miRNA features. These dimensions correspond to approximately 20% of those used in the individual cancer datasets, allowing the cross-cancer datasets to use a common shared feature space while preserving larger feature spaces for the individual cancer analyses. Each cross-cancer dataset therefore preserves the same omics structure and file organization as the training datasets, enabling direct use by both classical ML and deep learning modules without any additional preprocessing.

#### Batch effect-related preprocessing considerations.

Because TCGA multiomics data may contain technical heterogeneity arising from differences in collection centers, sample processing, and measurement pipelines, we adopted several practical measures during dataset construction to reduce potential batch-related variation. First, normalization was applied during preprocessing for each omics type. Second, for each omics modality, samples were restricted to a consistent measurement platform whenever possible. For example, DNA methylation data were uniformly collected from the Illumina HumanMethylation450 platform. These steps were intended to improve data consistency and reduce potential batch effects at the dataset level.

### ML module design

**Fig. 3.**
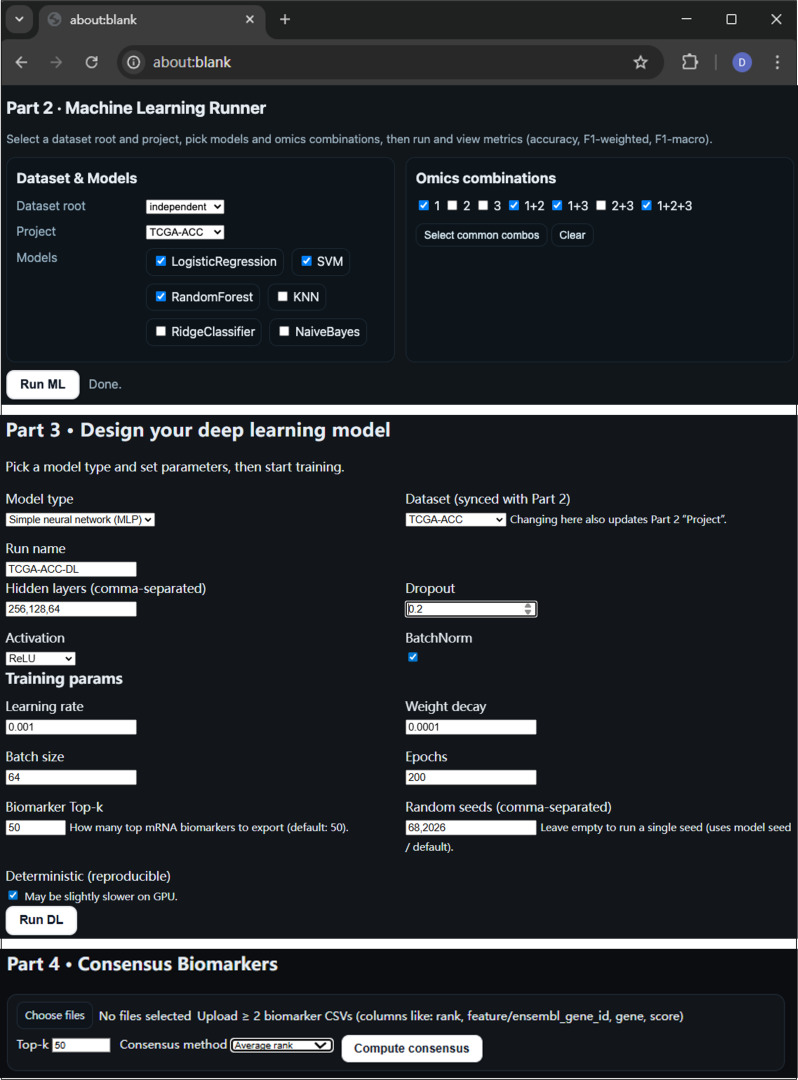
Preview of the CancerSubtypeXplore tool interface—parts 2 to 4. The integrated web interface provides modules for machine learning benchmarking, customizable deep learning model design, and consensus biomarker analysis.

The ML module in CancerSubtypeXplore serves as a reference benchmarking stage to establish baseline predictive performance on the curated multiomics datasets (Fig. 3, part 2).

This step enables researchers to quantitatively evaluate how well classical models can separate cancer subtypes under the same preprocessing and feature configuration, providing a meaningful context for the subsequent deep-learning-based biomarker discovery.

#### Data input and structure

For each TCGA project processed through the dataset module, 3 omics matrices (mRNA, DNA methylation, and miRNA) were used as inputs. Each matrix$\;X^{\left(k\right)}\in {\mathbb{R}}^{n\times p_{k}}$ was normalized and aligned as described earlier, where n denotes the number of samples and $p_{k}$ denotes the number of retained features in modality k.

To maintain comparability across methods, each ML model was trained independently on concatenated multiomics features

X=XmRNA‖XMeth‖XmiRNA, (4)

and evaluated using identical train/test partitions (70/30).

#### Model selection and implementation

Six representative supervised algorithms (Table 1) were implemented. These methods were selected to cover a range of inductive biases from linear discriminants to nonlinear ensemble learners. Thus, it provides diverse perspectives on model complexity, interpretability, and generalization behavior.

**Table 1.** Summary of classical machine learning algorithms integrated in CancerSubtypeXplore

Algorithm	Core principle	Main advantages
Logistic regression	Linear discriminative model using regularized regression	Simple and interpretable baseline
Support vector machine (SVM)	Margin maximization with kernel mapping	Effective in high-dimensional data
Random forest	Ensemble of decision trees with bagging	Robust to noise and feature correlation
*K*-nearest neighbors (KNNs)	Instance-based classification by distance	Nonparametric, captures local structure
Ridge classifier	Linear regression with L2 regularization	Stable and resistant to overfitting
Naïve Bayes	Probabilistic inference under feature independence	Fast and suitable for sparse data

The included ML models were selected as representative baseline classifiers covering different classifier families, including linear models, margin-based learning, ensemble learning, distance-based learning, and probabilistic classification, to support broad and interpretable benchmarking.

These models were evaluated using the default hyperparameter settings provided by the corresponding implementations in the scikit-learn library. No exhaustive grid search or dataset-specific hyperparameter tuning was performed. Therefore, the reported ML results should be interpreted as uniform default baseline references for comparison within the platform, rather than the fully optimized performance upper bound of each method.

The included ML models were selected as representative baselines covering different classifier families, including linear, distance-based, probabilistic, and ensemble methods, to support broad and interpretable benchmarking.

#### Evaluation metrics

Each model was evaluated using the same stratified train/test splits generated in the dataset module.

The main performance metric was classification accuracy (ACC), accompanied by F1-macro and F1-weighted scores to account for class imbalance.

Formally, for each project c with subtype classes $Y_{c}=$ $\left\{1,\;2,\;\ldots ,\;K_{c}\right\},$

$$\begin{array}{c} \mathrm{ACC}=\frac{1}{N}\sum_{i=1}^{N}I\left(\hat{y_{i}}=y_{i}\right),F1_{\text{macro}}=\frac{1}{K_{c}}\sum_{k=1}^{K_{c}}\frac{2P_{k}R_{k}}{P_{k}+R_{k}},\\ F1_{\text{weighted}}=\sum_{k=1}^{K_{c}}\frac{n_{k}}{N}\frac{2P_{k}R_{k}}{P_{k}+R_{k}} \end{array}$$ (5)

where Nis the total number of test samples, ${\hat{y}}_{i}\;$and $y_{i}\;$denote the predicted and true subtype labels for sample i, $P_{k}\;$and $R_{k}$ are the precision and recall for class k, $n_{k}\;$is the number of samples in class k, and $K_{c}\;$represents the total number of subtypes in project c. These metrics were consistently applied to evaluate not only the classical ML models but also all subsequent deep learning architectures described in later sections.

### Design your deep learning model module

This module in CancerSubtypeXplore provides users with a flexible environment for designing, training, and benchmarking neural network architecture without requiring any programming expertise. While the classical ML models serve as fixed benchmark for reference, this module allows researchers to build customized deep learning pipelines, explore the effect of architectural choices, and identify nonlinear patterns that might be inaccessible to traditional classifiers.

All models are implemented in PyTorch and are fully integrated with the dataset and evaluation components, ensuring end-to-end reproducibility.

#### Overview and design

Users can select among 3 neural network prototypes: (a) a simple linear baseline, (b) a multilayer perceptron (MLP), and (c) a self-attention model, each of which exposes configurable hyperparameters through an interactive interface (Fig. 3, part 3).

The goal of this module is not to enforce a single optimal network but rather to allow dynamic exploration of architecture and to compare their performance under consistent training conditions.

#### Model architecture

##### Simple linear (baseline)

The simplest model acts as a fully connected linear classifier:

$$\hat{y}=\text{Softmax}\left(\mathrm{XW}+b\right)$$ (6)

where$\;X\in {\mathbb{R}}^{n\times p}$ represents the concatenated multiomics feature vector, and W and *b* are trainable parameters.

This baseline defines a purely linear decision boundary, serving as the lower-complexity reference for subsequent nonlinear networks.

##### Multilayer perceptron

The MLP extends the linear model by introducing L hidden layers with user-specified dimensions and activation functions:

$$\begin{array}{c} \begin{array}{c} h_{1}=f\left(W_{1}X+b_{1}\right),h_{l}=f\left(W_{l}h_{l-1}+b_{l}\right),\hat{y}=\text{Softmax}\left(W_{L}h_{L-1}+b_{L}\right), \end{array} \end{array}$$ (7)

where $f\left(\cdot \right)$ can be rectified linear unit (ReLU), Gaussian error linear unit, exponential linear unit, or LeakyReLU.

Dropout and batch normalization are optionally applied between layers to improve regularization and convergence stability.

Users can flexibly specify hidden layer sizes as comma-separated values (e.g. “128, 64, 32”), while the training parameters (learning rate, weight decay, batch size, and epochs) remain consistent with those used in the baseline models for comparability.

##### Self-attention model

To capture dependencies between omics features that may span long-range relationships, we implemented a simplified self-attention model analogous to a transformer encoder.

Each block computes the following:

$$\text{Attention}\left(Q,\;K,\;V\right)=\text{Softmax}\left(\frac{QK^{⊤}}{\sqrt{d}}\right)V,$$ (8)

where $Q,K,\text{and}\;V\in {\mathbb{R}}^{d\times n}\;$are linear projections of the input and d is the latent dimensionality.

The network comprises $n_{\text{layers}}\;$encoder blocks, each with $n_{\text{heads}}\;$attention heads and a feedforward expansion multiplier $m_{\mathrm{ff}}$. This model enables the learning of feature-to-feature interactions and omics-specific dependencies in a data driven manner.

In the current version, simple baseline architectures were intentionally adopted to demonstrate the usability and configurability of the platform; more advanced architectures can be incorporated in future extensions.

#### Training protocol

Each model type is initialized with task-agnostic default hyperparameters to ensure comparable starting conditions while allowing user customization via the web interface.

1. MLP. The default configuration uses 3 hidden layers with dimensions [256, 128, 64], dropout = 0.2, ReLU activation, and batch normalization enabled.
2. Self-attention encoder. The default configuration adopts a lightweight transformer encoder with a model dimension of 32 (d_model = 32), 2 attention heads (n_heads = 2), 2 encoder layers (n_layers = 2), a feedforward expansion ratio of 2 (ff_multiplier = 2), and a dropout rate of 0.1.
3. Optimization scheme. All models are trained using the Adam optimizer with learning rate *η* = 10^−3^, weight decay *λ* = 10^−4^, batch size *B* = 64, and maximum epochs *E* = 200. Feature standardization (standardize = true) and balanced sampling (balanced = true) are applied by default.

Users can freely adjust these parameters through the interface, and the final results are displayed as ACC–F1 scatter plots and summary tables for direct performance comparison. In the current version, hyperparameter adjustment is performed in a practical user-guided manner, primarily based on manual or heuristic exploration rather than exhaustive grid search. This design is intended to support flexible benchmarking and exploratory analysis in an accessible no-code setting. Table 2 summarizes the adjustable parameters available for each model type.

**Table 2.** Configurable hyperparameters of deep learning models in CancerSubtypeXplore

Model type	Key architecture parameters	Regularization/training parameters
Simple linear (baseline)	–	Learning rate, weight decay, batch size, epochs
Multilayer perceptron (MLP)	Hidden layer sizes (comma-separated), activation function, dropout rate, batch normalization toggle	Learning rate, weight decay, batch size, epochs
Self-attention	Latent dimension $d_{\text{model}}$, number of layers $n_{\text{layers}}$, number of heads $n_{\text{heads}}$, feedforward multiplier $m_{\mathrm{ff}}$, dropout	Learning rate, weight decay, batch size, epochs

#### Evaluation protocol

Each dataset is divided into training and test sets according to a fixed 70/30 split strategy, with the test set reserved exclusively for final evaluation. This split ratio was adopted because several datasets contained fewer than 100 samples after preprocessing. Under these conditions, the 70/30 design retained most samples for model training while preserving a test set of at least 20 samples for final performance assessment. To avoid data leakage, all preprocessing, feature filtering, and hyperparameter adjustment steps are conducted using the training data only, and no information from the test set is used during model development. The held-out test set is not involved in hyperparameter tuning, architecture selection, or any other model selection step.

For both classical ML models and deep learning models, the same train/test split is applied within each dataset to ensure fair comparison. Model performance is evaluated using ACC, F1-weighted, and F1-macro, which together reflect overall classification performance and robustness under class imbalance. For binary classification tasks, the platform additionally outputs the receiver operating characteristic (ROC) curve and the area under the ROC curve as complementary threshold-independent evaluation metrics. The final results are summarized in tables and visualized in the platform for direct comparison across datasets and model types.

To improve reproducibility, explicit random seed control is incorporated into the training pipeline. The random seed can be specified by the user and is consistently applied to data splitting and model initialization, enabling reproducible experiments under controlled settings.

To reduce the risk of overfitting in deep learning models under small-sample settings, an early stopping strategy is applied during training. In addition, the platform outputs loss curves during the training process, allowing users to monitor optimization behavior and visually inspect potential overfitting. Hyperparameter adjustment is performed in a practical user-guided manner, primarily based on manual or heuristic exploration rather than exhaustive grid search. This design is intended to support flexible benchmarking and exploratory analysis while maintaining a clear separation between model development and final performance assessment.

In addition, the platform supports multiple result visualization functions, including ROC-based evaluation, training–monitoring outputs, and biomarker-based exploratory analysis, with representative examples shown in Figs. S1 to S3.

#### Integration with the pipeline

All trained deep learning models are seamlessly linked with the biomarker analysis module. After training, the feature attribution or learned weight matrices are automatically parsed to generate ranked feature importance lists, which are then aggregated across models to identify robust cross-model biomarkers. In this way, the deep learning module not only achieves higher predictive ACC than the classical ML baselines but also provides interpretable insights into omics-level feature contributions.

### Biomarker analysis module

The biomarker analysis module represents the final stage of the CancerSubtypeXplore workflow, bridging model performance evaluation with biological interpretation (Fig. 3, part 4).

Its primary purpose is to identify robust, cross-model, and cross-cancer biomarkers that consistently contribute to accurate subtype classification across diverse algorithms. This module aggregates feature importance information derived from the customizable deep learning models, computes their overlaps, and ranks candidate biomarkers according to their cross-model or cross-cancer stability.

Each trained deep learning model (linear baseline, MLP, or self-attention network) produces a set of parameters or latent representations encoding feature importance. After training, the system automatically computes contribution scores for all input features within each omics layer. These scores are standardized and ranked to identify potential biomarkers associated with cancer subtypes.

Biomarker candidates were selected using a fixed topk rule, with the top 50 ranked features retained for each model. The platform allows the user to specify the value of kthrough the interface, so that the number of reported biomarkers can be adjusted according to different analysis needs. Thus, the biomarker output is based on contribution score ranking with a user-configurable topk threshold, while the default setting remains k=50.

#### Feature contribution calculation

In this study, we estimate feature contribution using a gradient-based saliency score computed after training. Let $f\left(x\right)\in {\mathbb{R}}^{C}$denote the model logits for C classes. For each sample $\left(x,\;y\right)$, we compute the absolute input gradient of the ground-truth class logit with respect to each input feature:

$$s_{j}\left(x,\;y\right)=|\frac{\partial f_{y}\left(x\right)}{\partial x_{j}}|.$$ (9)

The feature-level contribution score is obtained by averaging over the held-out set:

$$C_{j}=E_{\left(x,\;y\right)∼D}\left[s_{j}\left(x,\;y\right)\right].$$ (10)

In implementation, we accumulate $|\partial f_{y}\left(x\right)/\partial x|$across minibatches and divide by the number of samples. For multiomics inputs formed by concatenation, we compute $C_{j}\;$for all features and then extract the mRNA segment to report the top-ranked candidate biomarkers. We standardize features using statistics computed on the training set and apply the same transformation to the held-out set.

This score measures local sensitivity and may be affected by feature scaling and correlated inputs; therefore, we interpret the resulting biomarkers as stable predictive features rather than causal drivers.

#### Cross-model biomarker discovery

To enhance robustness and mitigate model-specific bias, CancerSubtypeXplore performs cross-model consensus analysis. After each deep learning architecture produces its ranked feature list, features are aggregated according to their frequency of occurrence among the top k ranked positions across all trained models. Let $F^{\left(m\right)}\;$denote the topk feature set of model m; then, the final consensus biomarker frequency for feature iis computed as

$$f_{i}=\frac{1}{M}\sum_{m=1}^{M}I\left(i\in F^{\left(m\right)}\right),$$ (11)

where M is the number of models evaluated and $I\left(\cdot \right)$ is the indicator function.

Features are then sorted by $f_{i}\;$in descending order to identify those consistently highlighted across models. This frequency-based strategy helps isolate biomarkers that are stable and reproducible under different architectures or hyperparameter settings.

#### Cross-cancer biomarker discovery

Beyond model-level comparison, CancerSubtypeXplore also supports cross-cancer biomarker consensus analysis to explore molecular signatures shared across different tumor types.

Users may select any subset of TCGA datasets (e.g., TCGA-BRCA, TCGA-COAD, TCGA-HNSC, TCGA-LIHC, TCGA-PCPG, TCGA-STAD, and TCGA-UCEC) and extract top-ranked mRNA features from the best-performing model of each dataset.

Given the top *k* feature set $F^{\left(c\right)}\;$from cancer type c, the cross-cancer occurrence frequency of feature i is computed as

$$f_{i}^{\left(\text{cancer}\right)}=\frac{1}{C}\sum_{c=1}^{C}I\left(i\in F^{\left(c\right)}\right),$$ (12)

where Cis the number of cancers selected and $I\left(\cdot \right)$ is the indicator function.

Features with higher $f_{i}^{\left(\text{cancer}\right)}\;$values are considered cross-cancer biomarkers, representing genes consistently associated with subtype discrimination across distinct tumor contexts.

This function enables users to identify stable, potentially pan-cancer molecular signals, even when applying the analysis to only a subset of available datasets.

#### Biological interpretation and output

The final ranked biomarker list for each cancer type includes the corresponding feature ID (gene, CpG probe, or miRNA), its normalized contribution score, and its frequency across models. These tables are exported as .csv files, facilitating downstream enrichment or pathway analysis using tools such as g:Profiler [19], DAVID [20], or Enrichr [21]. In addition, summary statistics (mean contribution and rank variance) are visualized to assess the stability of the identified biomarkers across independent model runs.

## Results

### Benchmarking of classical ML models across 17 TCGA datasets

To establish a rigorous benchmark prior to the deep learning modules, we evaluated 6 ML methods—logistic regression (LR), SVM, RF, *K*-nearest neighbors (KNNs), Ridge, and naïve Bayes (NB)—across 17 TCGA datasets. Performance was assessed using ACC, F1-weighted, and F1-macro. All models were trained under a unified preprocessing and evaluation protocol, ensuring fair comparability across methods and datasets.

**Fig. 4.**
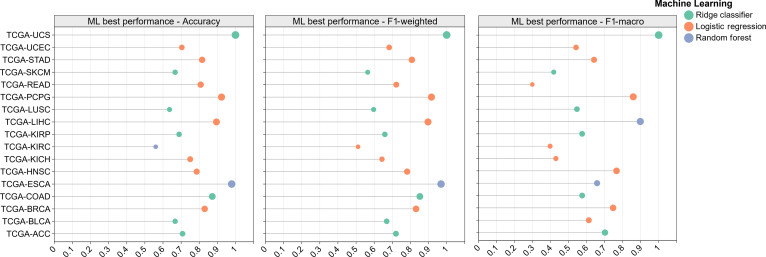
Best machine learning performance across 17 TCGA datasets. Each point represents the best-performing classical algorithm per dataset under 3 evaluation metrics (accuracy, F1-weighted, and F1-macro). Ridge classifier (Ridge), logistic regression, and random forest achieved the highest scores in different cancer types. This figure was drawn using ChiPlot (https://www.chiplot.online/) (accessed on 2025 October 9).

Comprehensive results for all 6 algorithms are reported in Table S2 and Figs. S4 to S6, whereas Fig. 4 visualizes, for each dataset and each metric, only the best performing algorithm to highlight the attainable ML performance envelope. Under this selection criterion, only 3 models—Ridge, LR, and RF—emerged as winners across the 17 datasets. SVM, KNN, and NB did not achieve the top score on any dataset/metric combination under our protocol, which explains their absence from the figure despite being fully benchmarked in Table S2.

The pattern of winners is consistent with the statistical properties of high-dimensional multiomics data. Ridge and LR (both L2-regularized linear decision functions) frequently topped ACC, F1-weighted, and F1-macro on datasets with many correlated features and moderate class imbalance, where explicit regularization stabilizes estimates and reduces variance. RF tended to win on datasets exhibiting nonlinear signal or feature label interactions, benefiting from bagging and feature subsampling that confer robustness to noisy or redundant variables. In contrast, KNN is adversely affected by the “curse of dimensionality”, NB suffers from the strong conditional independence assumption, and SVM (in our standard configuration) was competitive but did not surpass the winners once regularization and ensembling effects were accounted for.

Collectively, these results define the ML ceiling for each dataset and metric while preserving method-level transparency. They provide a principled reference point for the subsequent deep learning modules: Improvements beyond the Ridge/LR/RF’s performance indicate genuine modeling gains rather than artifacts of data handling or evaluation.

### Cross-model biomarkers in BRCA

Breast cancer is the most common malignant tumor worldwide [22]. In this study, we focused on BRCA, the most extensively studied and well characterized cancer, featuring abundant multiomics data and clearly defined molecular subtypes [23]. The TCGA-BRCA dataset was selected as a representative case to demonstrate our cross-model biomarker extraction framework. Moreover, numerous experimentally validated biomarkers have been reported in previous studies, providing a robust biological reference for assessing whether the biomarkers identified by our cross-model approach are biologically meaningful. Therefore, BRCA serves as an ideal benchmark for assessing the robustness, interpretability, and biological plausibility of the proposed consensus biomarker identification strategy.

In Table 3, model names such as MLP (128, 64) indicate a 2-hidden-layer MLP with hidden dimensions of 128 and 64, respectively, while self-attention (2, 2, 2, 32) refers to a transformer-style encoder configured with 2 attention heads, a feedforward expansion multiplier of 2, 2 encoder layers, and a model dimension of 32. Although self-attention architectures are regarded as state of the art, they consistently failed to outperform the ML baseline on the BRCA dataset despite extensive tuning. This limitation likely stems from a mismatch between parameter complexity and available sample size, the greater sensitivity of attention layers to optimization and regularization, and the fact that discriminative subtype signals in BRCA are relatively sparse across features. Under such conditions, simpler networks may generalize more effectively; therefore, we include one representative self-attention configuration below the ML baseline for completeness, while all other deep learning models listed achieved higher scores than the ML reference. Notably, the best-performing configurations did not always arise from integrating all 3 omics layers. Models using mRNA alone or mRNA + DNA methylation often surpassed those including miRNA, reflecting that adding weaker or noisier modalities can dilute signal strength, introduce batch heterogeneity, and increase dimensionality without improving discrimination. Accordingly, CancerSubtypeXplore allows systematic comparison across different omics combinations rather than if more data types necessarily yield better performance. To assess the effect of random initialization, we additionally tested representative BRCA models under multiple random seeds and provided the corresponding results in Table S3. The overall ranking trend and performance pattern were similar across seeds. We also evaluated whether the selected biomarkers retained subtype-related structure by generating principal-components-analysis-based visualizations using only the extracted biomarkers. As shown in Fig. S3 for the TCGA-BRCA case, the selected biomarkers showed separation among subtypes in the reduced feature space, providing a complementary visualization of the subtype-related information retained by these features. The corresponding cross-model consensus biomarkers are summarized in Table 4, representing features recurrent across at least 4 of 7 deep models (frequency ≥ 4); bolded genes denote biomarkers with prior peer-reviewed evidence supporting their association with BRCA.

**Table 3.** BRCA deep-model configurations and performance

Architecture	Omics	ACC	F1-weighted	F1-macro
MLP (256, 128, 64)	mRNA + DNA methylation + miRNA	0.830	0.825	0.710
MLP (256, 128, 64)	mRNA + DNA methylation	0.838	0.829	0.743
MLP (128, 64, 32)	mRNA + DNA methylation + miRNA	0.821	0.820	0.719
MLP (128, 64)	mRNA + DNA methylation + miRNA	0.834	0.838	0.762
MLP (128, 64)	mRNA + DNA methylation	0.834	0.835	0.769
Self-attention (2, 2, 2, 32)	mRNA	0.777	0.791	0.633
Simple linear neural network	mRNA + DNA methylation + miRNA	0.825	0.827	0.718

MLP, multilayer perceptron; Acc, accuracy; miRNA, microRNA.

**Table 4.** Breast cancer cross-model consensus biomarkers (frequency ≥ 4)

Biomarkers (with frequency)
***LMNB1*** (7), ***DOK1*** (7), ***MIR3156-2*** (5), *RNU6-1028P* (5), *C1QTNF7-AS1* (5), *TRBV12*- 1 (5), *GAPDHP63* (5), *PSPC1-AS2* (5), *PGA3* (4), *ASIC4-AS1* (4), *LENG8-AS1* (4), *MIR6810* (4), *PHB1P16* (4), *SMUG1P1* (4)

Bold biomarkers have peer-reviewed evidence linking them to breast cancer.

Notably, several consensus biomarkers, defined as the intersection of biomarkers identified across models, have prior support in the BRCA literature. *LMNB1 *is an important component of the nuclear backbone (frequency = 7) has been reported to be aberrantly expressed in breast cancer and may inactivate peroxisome proliferator-activated receptor signaling to promote the malignant development of breast cancer cells [24,25]. *DOK1 *(frequency = 7) exhibits reduced mRNA expression in breast tumor tissues and has been suggested act as a tumor suppressor by modulating the mitogen-activated protein kinase pathway through epidermal growth factor receptor signaling [26]. The mature *miR-3156-5p*, derived from the *MIR3156-2* (frequency = 5) locus, is implicated in regulating chemoresponse and growth in breast cancer [27,28]. Through our cross-model analysis, many of the high-frequency genes we identified have already been reported in previous studies. This demonstrates that our contribution-based aggregation strategy is not confined to any specific model architecture and can robustly capture BRCA-related biological characteristics.

### Cross-cancer biomarkers across 5 TCGA datasets

To provide representative exemplars for cross-cancer biomarker analysis, we selected 5 cross-cancer TCGA dataset: TCGA-BRCA, TCGA-COAD, TCGA-LIHC, TCGA-STAD, and TCGA-PCPG. The first 4 represent some of the most common, high-burden epithelial malignancies globally, as shown in GLOBOCAN cancer incidence and mortality statistics [29]. Meanwhile, PCPG is a comparatively rare neuroendocrine tumor with systemic endocrine/metabolic effects (e.g., catecholamine secretion), offering a unique lens into endocrine–cancer interactions across tumor types. We trained and tuned MLP models independently for each dataset. For each cancer type, hyperparameters such as the number of hidden layers, layer dimensions, and learning rate were adjusted until the model achieved performance surpassing its respective ML benchmark.

As summarized in Table 5, not all datasets favored the same architecture—while MLP (128, 64) provided optimal results in most cases, certain cohorts (e.g., STAD) required a deeper configuration (MLP (128, 64, 32) to stabilize learning. This variability reflects intrinsic differences in omics data complexity and subtype separability across cancers. After model convergence, the top-ranked features were extracted based on contribution scores, and their cross-cancer recurrence was analyzed. Features present in at least 2 of the 5 cancers were designated as cross-cancer (pan-cancer) biomarker candidates (Table 6).

**Table 5.** Performance of the best MLP per cancer (5 datasets)

Dataset	Architecture	Omics	ACC	F1-weighted	F1-macro
BRCA	MLP (128, 64)	mRNA + DNA methylation	0.834	0.835	0.769
COAD	MLP (128, 64)	mRNA + DNA methylation	0.872	0.861	0.598
LIHC	MLP (128, 64)	mRNA + DNA methylation	0.912	0.912	0.905
PCPG	MLP (128, 64)	mRNA + DNA methylation	0.923	0.923	0.868
STAD	MLP (128, 64, 32)	mRNA	0.816	0.806	0.630

MLP, multilayer perceptron; BRCA, breast invasive carcinoma; COAD, colon adenocarcinoma; LIHC, liver hepatocellular carcinoma; PCPG, pheochromocytoma and paraganglioma; STAD, stomach adenocarcinoma.

**Table 6.** Cross-cancer consensus biomarkers (frequency ≥ 2)

Biomarkers (with frequency)
***LMNB1*** (2), ***MUC4*** (2), ***DOK1*** (2), ***GRM8*** (2), *MIR1244-3* (2), *LENG8-AS1* (2), *TIGD7* (2), *IGLV3-13* (2)

Bold biomarkers have peer-reviewed evidence linking them to cancers.

Among the recurrently identified genes, *LMNB1* (frequency = 2) was detected again. This gene has been implicated in the development of multiple cancer types [30]. *MUC4 *(frequency = 2) is a membrane mucin frequently up-regulated in metastatic breast tumors and associated with aggressiveness and poorer outcomes, corroborating its appearance in multiple cancers [31–33]. Down-regulation of *DOK1* (frequency = 2) mRNA expression has been reported in various tumors, including hepatocellular carcinoma, colorectal cancer, ovarian cancer, and lung cancer. Moreover, emerging evidence suggests that *DOK1* may act as a tumor suppressor in breast cancer [25,34]. Finally, emerging evidence indicates that metabotropic glutamate receptor 8 (*GRM8*) (frequency = 2) is involved in the progression of multiple malignancies, including neuroblastoma, lung cancer, and glioma, and may also exert oncogenic activity in breast cancer [35,36].

### External validation and functional enrichment analysist

Among the biomarkers identified from the TCGA-BRCA dataset, several genes, including *LMNB1, DOK1, MIR3156-2, RNU6-1028P, C1QTNF7-AS1, TRBV12-1, GAPDHP63, PSPC1-AS2, PGA3, ASIC4-AS1, LENG8-AS1, MIR6810, PHB1P16*, and *SMUG1P1*, were repeatedly selected across multiple runs, indicating repeated selection under the current analysis setting (Table 4). Direct overlap between the TCGA-BRCA biomarker list and the biomarkers extracted from the GEO-GSE96058_BRCA dataset was limited. However, despite the platform-dependent differences in the exact gene lists, partial overlap was still observed at the functional level. For example, *DOK1* is a docking/adaptor protein involved in receptor-mediated signal transduction, whereas TRBV12-1 belongs to the T cell receptor β variable locus; therefore, these TCGA-BRCA biomarkers are functionally related to signaling- and immune-associated categories, which partially overlap with the receptor- and immune-associated features identified in the GEO-GSE96058_BRCA-derived biomarkers [34,37]. These results indicate that, although the exact biomarkers differed substantially between datasets, the selected biomarkers from the 2 datasets showed partial overlap in functional annotation.

Because the original TCGA-BRCA features were largely represented by Ensembl gene identifiers, many biomarkers could not be directly mapped to standard gene symbols, which limited subsequent enrichment analysis. Therefore, we additionally performed pathway enrichment analysis using the biomarkers extracted from the GEO-GSE96058_BRCA dataset (Fig. S7). The enriched terms were mainly associated with developmental and epithelial-related processes, including developmental cell lineages, developmental lineages of the mammary gland, and epithelial cell migration. Additional enrichment was observed in hormone regulation, matrisome-associated processes, cell surface interactions at the vascular wall, and the complement system. Together, these results place the GEO-GSE96058_BRCA-derived biomarkers in a functional context related to mammary developmental programs, epithelial behavior, hormone-related regulation, and tumor-microenvironment-associated processes [38–40].

## Discussion

The present study introduces CancerSubtypeXplore, a modular web-based platform designed to streamline multiomics cancer subtype analysis and biomarker discovery. By integrating standardized TCGA datasets, a suite of classical ML algorithms, customizable deep learning architectures, and a contribution-based interpretability module, the system enables reproducible exploration of multiomics data without extensive coding. The benchmarking results across 17 TCGA datasets demonstrate that while traditional ML models provide a baseline level of subtype discrimination, deep learning architectures substantially improve predictive ACC and F1 metrics, particularly when multiple omics layers are integrated.

Compared with existing frameworks such as MOVICS [11], DeepProg [6], and MLOmics [13], CancerSubtypeXplore is primarily designed as a modular benchmarking and exploration platform rather than a new predictive method intended to outperform existing multiomics integration frameworks. Its main contribution lies in providing a unified and practical environment for multiomics subtype analysis. Specifically, the platform integrates and standardizes multiomics datasets in a consistent format for comparative analysis and supports no-code model design, benchmarking, and hyperparameter adjustment, thereby lowering the barrier for users to explore different ML and deep learning strategies.

The cross-model analysis using BRCA as an example revealed several high-frequency biomarkers—*LMNB1*, *DOK1*, and *MIR3156-2*—all supported by independent experimental studies linking them to tumor progression, signaling regulation, and chemoresistance [24–28]. Their consistent recurrence across architecture indicates that CancerSubtypeXplore effectively captures biologically meaningful signals rather than model-specific noise. The subsequent cross-cancer study identified additional recurrent genes such as *MUC4*, *GRM8*, and *LMNB1*, reflecting molecular pathways shared among epithelial malignancies [30–36]. These findings suggest that the contribution frequency framework may reveal both tissue-specific and pan-cancer mechanisms, offering a unified view of tumorigenesis across diverse contexts.

A key strength of CancerSubtypeXplore is its modularity and transparency: Users can customize model depth, activation functions, and omics combinations. Moreover, the interactive visualization of contribution-based feature importance provides an intuitive means for biomedical researchers to inspect model decisions. Nonetheless, several limitations remain. First, the current implementation relies on TCGA bulk-level omics and may not directly generalize to single-cell or spatial data. In addition, although normalization and platform consistency filtering were applied during preprocessing, explicit batch effect assessment and dedicated batch correction were not included in the current workflow. Therefore, residual technical heterogeneity arising from differences in collection centers, sample processing, or measurement pipelines may still influence classification performance and biomarker stability. Incorporating optional batch correction and cross-platform harmonization will be an important direction for future development. We also note that the current evaluation strategy is limited by the use of a fixed 70/30 train/test split, without repeated runs or cross-validation, partly because some datasets contain relatively small sample sizes. In addition, the relatively small cohort sizes may increase the risk of unstable performance and overfitting in deep learning models, although early stopping and loss curve outputs were incorporated in the current implementation to help monitor this risk. Second, while the contribution frequency method highlights stable biomarkers, it does not yet infer causality or regulatory directionality. Integrating pathway-level enrichment and network-based validation would further strengthen biological interpretation. Finally, model training currently uses static hyperparameter grids; future versions could incorporate Bayesian or reinforcement-learning optimization to adaptively explore architecture space.

Looking ahead, CancerSubtypeXplore is inherently extensible. In the future, users can easily incorporate additional cancer types or explore cross-cancer relationships by organizing new datasets following the same standardized data structure, thereby enabling subtype prediction and biomarker extraction without additional implementation effort. Beyond this, the framework could be further extended toward multicenter clinical data integration and cross-platform normalization to mitigate batch effects and enable joint modeling across studies [41–43]; The deep learning module can also be extended in future versions to include additional architectures, such as autoencoder-based representation learning models and other advanced methods, to support more complex scenarios including incomplete multiomics settings. It could also be extended toward automated annotation of literature-validated biomarkers by linking to curated oncology knowledge bases and literature-mined resources [44–47]. Coupling this framework with explainable artificial intelligence, such as integrated gradients, SHapley Additive exPlanations, or attention heatmaps, will further enhance interpretability and facilitate clinical translation of deep multiomics modeling [48,49].

## Conclusion

CancerSubtypeXplore provides an integrated, interpretable, and reproducible environment for multiomics cancer subtype analysis. By unifying standardized data curation, model benchmarking, customizable deep learning design, and consensus-based biomarker extraction, the platform bridges the methodological gap between high performance modeling and biological interpretability. Across 17 TCGA datasets, deep learning models consistently outperformed classical ML baselines, and consensus analysis successfully recovered biomarkers with literature-supported links to tumorigenesis. The cross-cancer results further highlight shared molecular patterns that transcend individual cancer types.

CancerSubtypeXplore establishes a transparent computational foundation for the next generation of integrative oncology tools empowering biomedical researchers to move from complex multiomics data toward stable, biologically validated, and clinically relevant insights.

## Data Availability

CancerSubtypeXplore software is available at https://github.com/KelvinJin08/CancerSubtypeXplore. The full source code is publicly available in this repository. In addition, the curated datasets used in this study, together with the relevant instructions for access and use, are also provided through the same repository.
