# Supplementary material for: CancerSubtypeXplore: A Modular Platform for Multiomics Cancer Subtype Prediction and Biomarker Consensus Discovery
Source: Comput Struct Biotechnol J. 2026 Apr 13;35(1):0030. doi: 10.34133/csbj.0030 (PMC13072625; doi:10.34133/csbj.0030)
Supplement: Supplementary 1 — Figs. S1 to S7 Tables S1 to S3 Movie S1 [file csbj.0030.f0030.zip › Supplementary_Information_0312.pdf]

To provide representative examples of the outputs generated by CancerSubtypeXplore, we included several illustrative result figures from different analysis modules. These examples show typical visualizations produced by the platform, including model evaluation, training monitoring, and biomarker-based exploratory analysis. Specifically, Figure S1 presents a representative ROC curve for binary classification using TCGA-UCS, Figure S2 shows the training loss curve and early stopping behaviour for TCGA-BRCA, and Figure S3 illustrates PCA projection based on the top 50 biomarkers identified in TCGA-BRCA, together with KMeans clustering. These example figures are provided to demonstrate the diversity of analysis outputs supported by the platform.

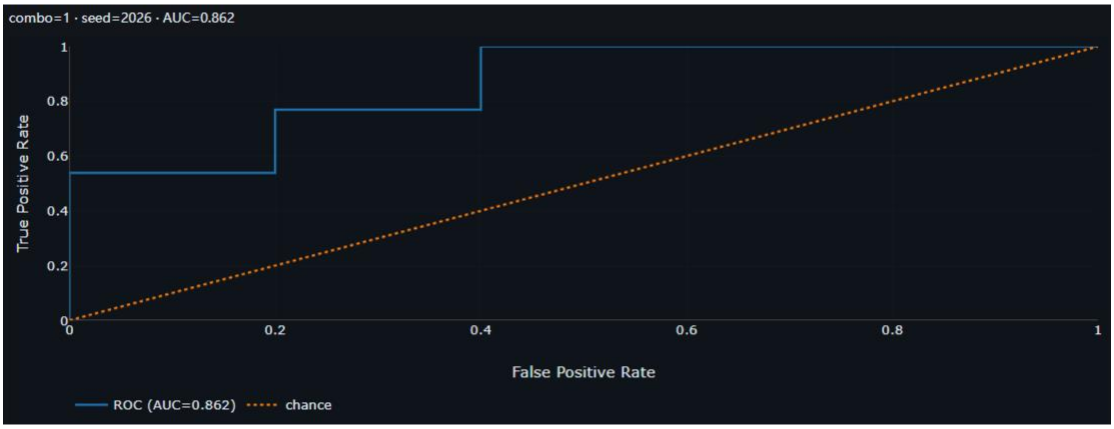

Figure S1. ROC curve for binary classification (TCGA-UCS).

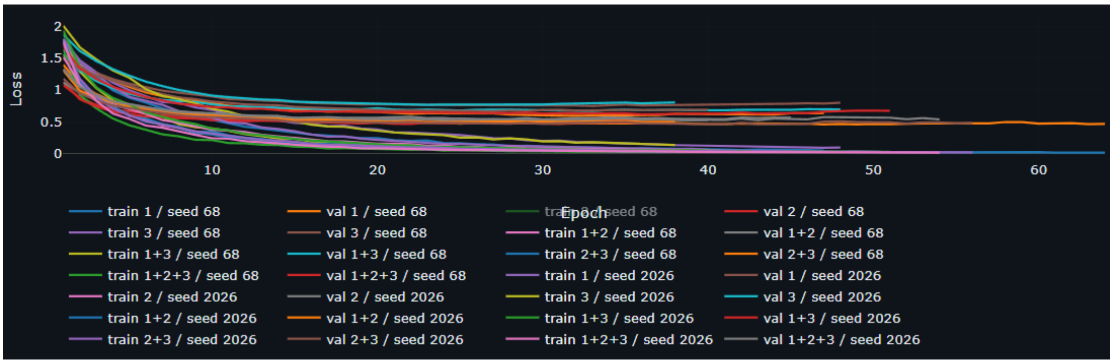

Figure S2. Loss curve and early stop (TCGA-BRCA).

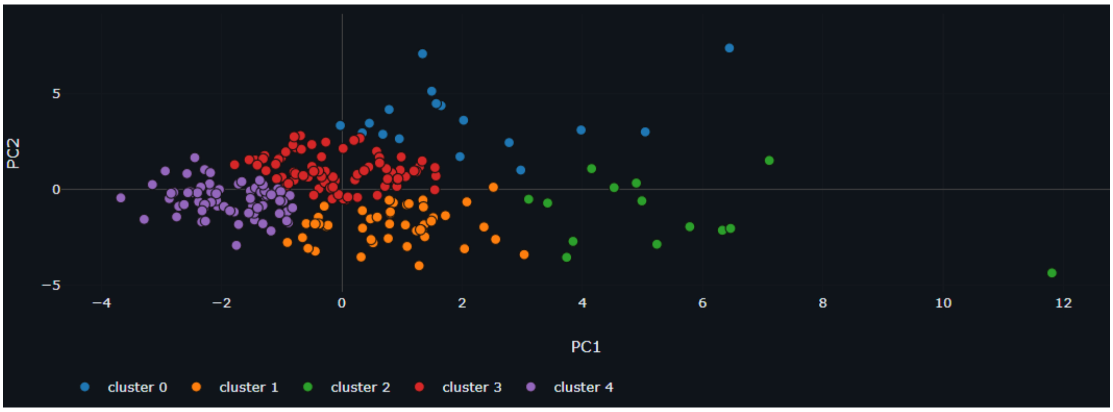

Figure S3. PCA using only Top-50 biomarkers · Explained Variance Ratio (EVR) = 0.088,

0.065 · KMeans K=5 (TCGA-BRCA)

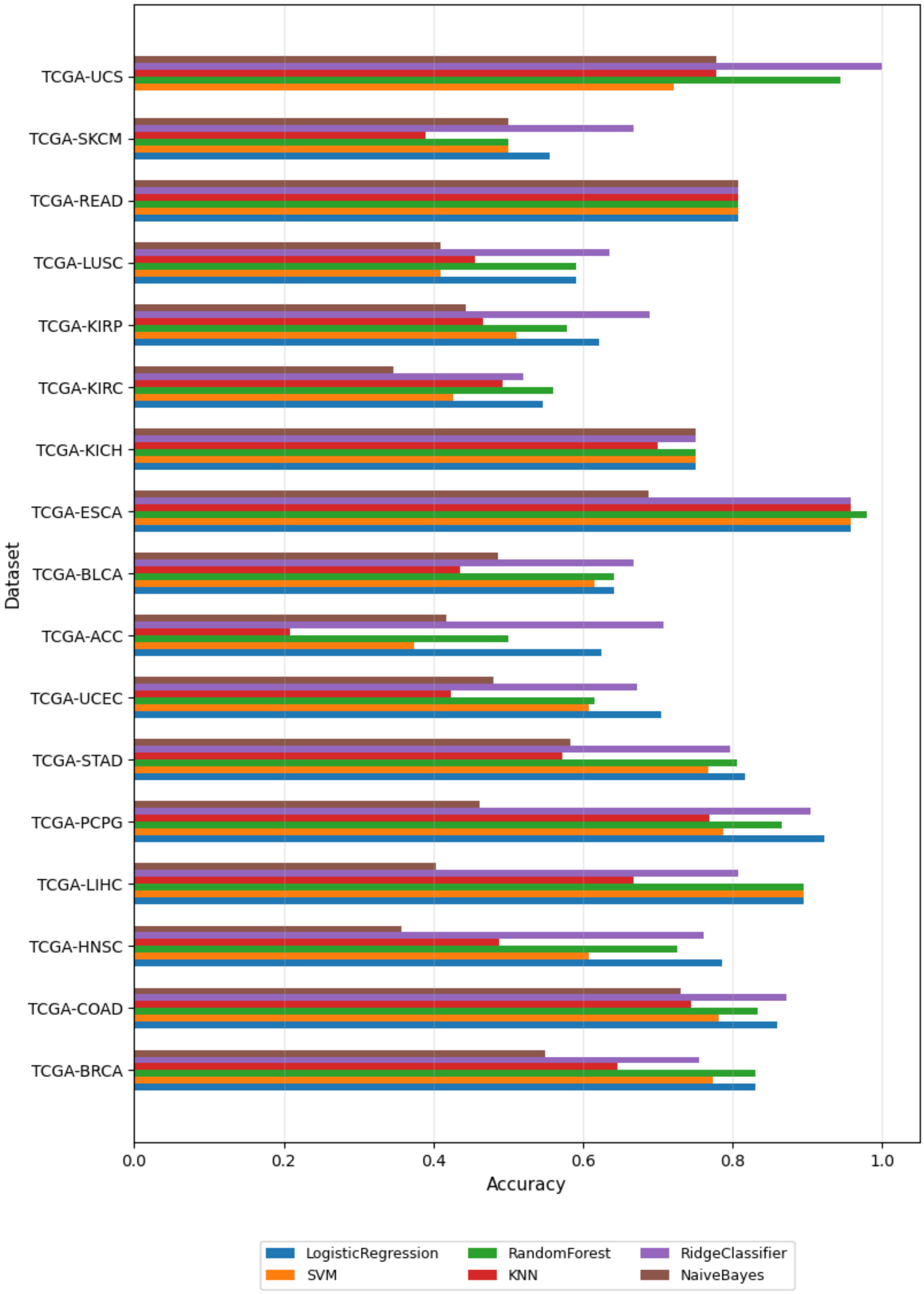

Figure S4. Accuracy of classical machine-learning methods across 17 TCGA datasets based on the complete benchmark results in Table S2.

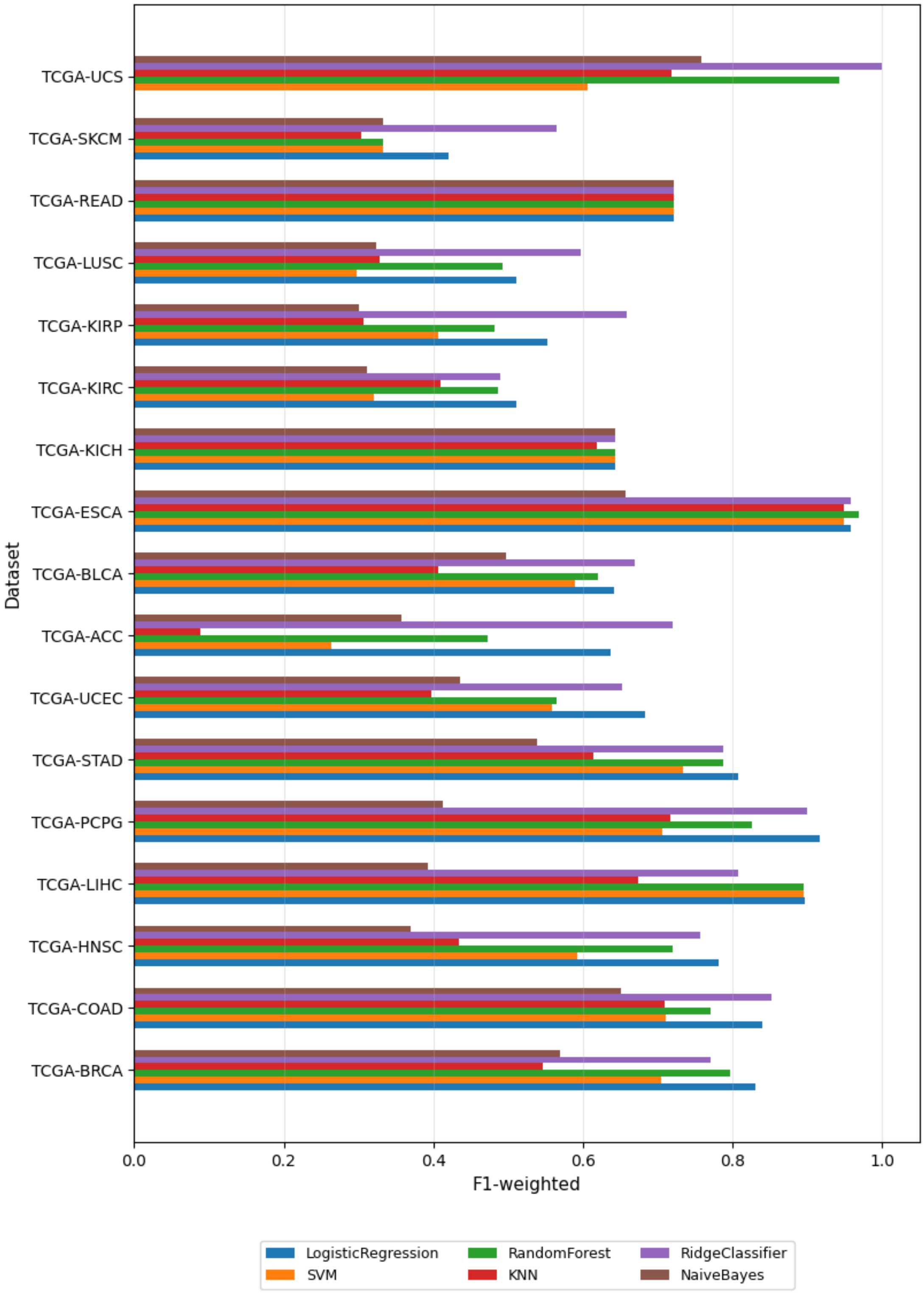

Figure S5. F1-weighted of classical machine-learning methods across 17 TCGA datasets based on the complete benchmark results in Supplementary Table S2.

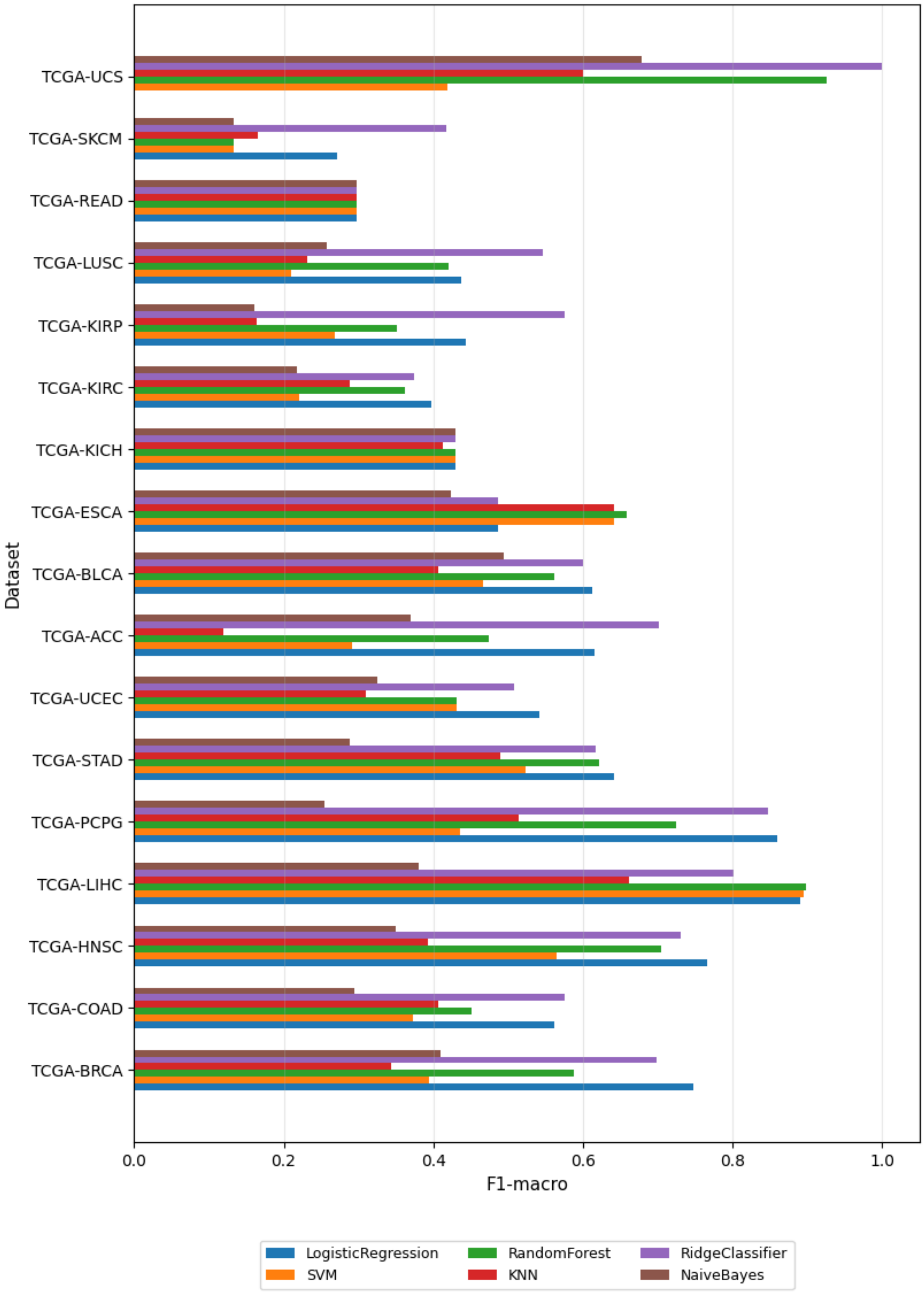

Figure S6. F1-macro of classical machine-learning methods across 17 TCGA datasets based on the complete benchmark results in Supplementary Table S2.

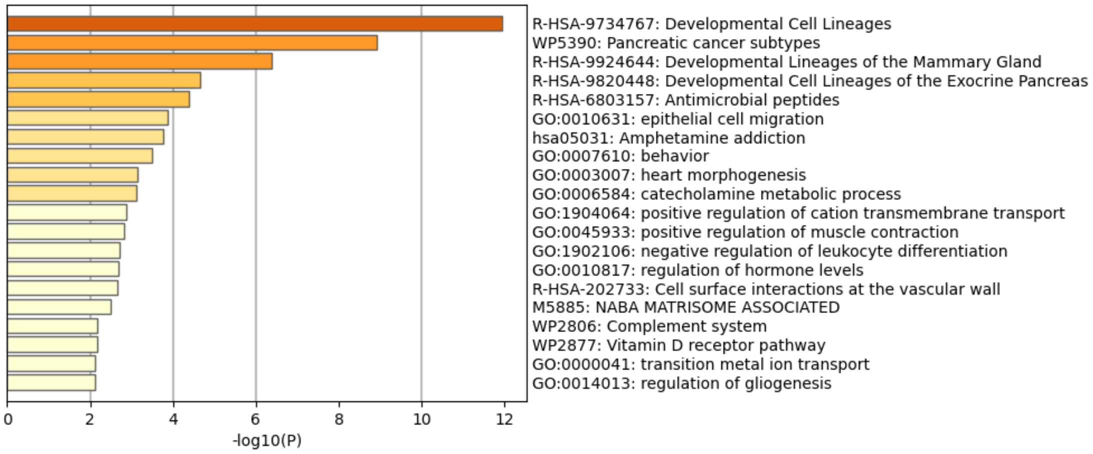

Figure S7. Bar graph of enriched terms across biomarkers (GEO-GES96058_BRCA), colored by p-values, using top 150 biomarkers.

Enrichment analysis of the biomarkers extracted from the GEO-GSE96058_BRCA dataset showed that these genes were primarily involved in developmental, differentiation, and epithelial-related processes. Highly enriched terms included Developmental Cell Lineages, Developmental Lineages of the Mammary Gland, and epithelial cell migration, together with pathways related to hormone regulation, matrisome-associated processes, vascular wall interactions, and the complement system. These findings provide a broader functional context for the externally validated BRCA biomarkers and suggest that, although the exact biomarker genes may differ across datasets, they may still converge on partially shared biological themes at the pathway level.

Table S1: Summary of 17 TCGA datasets (10 independent and 7 cross-cancer datasets) and one example external dataset

Dataset	Categories	Train Set	Test Set
**Independent datasets**			
TCGA-KIRC	KIRC.1	175	75
	KIRC.2		
	KIRC.3		
	KIRC.4		
	KIRC.NA		
TCGA-READ	GI.CIN	60	26
	GI.GS		
	GI.HM-SNV		
	GI.MSI		
TCGA-ESCA	GI.CIN	111	48
	GI.HM-SNV		
	GI.ESCC		
	GI.GS		
	GI.MSI		
TCGA-KICH	KICH.Eosin.0	45	20
	KICH.Eosin.1		
TCGA-UCS	UCS.1	39	18
	UCS.2		
TCGA-KIRP	KIRP.C2a	102	45
	KIRP.C1		
	KIRP.C2c - CIMP		
	KIRP.C2b		
TCGA-ACC	ACC.CIMP-high	54	24
	ACC.CIMP-low		
	ACC.CIMP-intermediate		
TCGA-SKCM	SKCM.BRAF	45	18
	SKCM.NF1		
	SKCM.RAS		
	SKCM.Triple Wild-Type		
	SKCM.NA		
TCGA-BLCA	BLCA.Luminal	88	39
	BLCA.Basal		
	BLCA.Neuroendocrine		
	BLCA.Stroma-rich		
TCGA-LUSC	LUSC.Luminal	51	22
	LUSC.Basal		
	LUSC.Classical		
	LUSC.Secretory		
**Cross-cancer datasets**			
TCGA-BRCA	BRCA.Normal	532	229
	BRCA.LumA		
	BRCA.Her2		
	BRCA.LumB		
	BRCA.Basal		

Continued on next page

Dataset	Categories	Train Set	Test Set
TCGA-PCPG	PCPG.Kinase signaling	118	52
	PCPG.NA		
	PCPG.Wnt-altered		
	PCPG.Pseudohypoxia		
	PCPG.Cortical admixture		
TCGA-LIHC	LIHC.iCluster:1	130	57
	LIHC.iCluster:2		
	LIHC.iCluster:3		
	LIHC.NA		
TCGA-COAD	GI.CIN	182	78
	GI.MSI		
	GI.GS		
	GI.HM-SNV		
TCGA-UCEC	UCEC.CN_HIGH	289	125
	UCEC.CN_LOW		
	UCEC.POLE		
	UCEC.NA		
	UCEC.MSI_H		
TCGA-STAD	STAD.EBV	238	103
	STAD.MSI		
	STAD.Genome Stable		
	STAD.Chromosomal Instability		
	STAD.NA		
TCGA-HNSC	HNSC.Atypical	193	84
	HNSC.Basal		
	HNSC.Classical		
	HNSC.Mesenchymal		
**Example external dataset**			
GEO-GSE96058_BRCA	BRCA.Normal	2292	981
	BRCA.LumA		
	BRCA.Her2		
	BRCA.LumB		
	BRCA.Basal		

Table S2: Classical machine-learning performance across 17 TCGA datasets using integrated multi-omics features.

Dataset	Machine Learning Method	Accuracy	F1-weighted	F1-macro
TCGA-UCS	SVM	0.722	0.606	0.419
TCGA-UCS	RandomForest	0.944	0.942	0.926
TCGA-UCS	KNN	0.778	0.719	0.600
TCGA-UCS	RidgeClassifier	**1.000**	**1.000**	**1.000**
TCGA-UCS	NaiveBayes	0.778	0.758	0.679
TCGA-SKCM	LogisticRegression	0.556	0.420	0.272
TCGA-SKCM	SVM	0.500	0.333	0.133
TCGA-SKCM	RandomForest	0.500	0.333	0.133
TCGA-SKCM	KNN	0.389	0.304	0.166
TCGA-SKCM	RidgeClassifier	**0.667**	**0.564**	**0.417**
TCGA-SKCM	NaiveBayes	0.500	0.333	0.133
TCGA-READ	LogisticRegression	**0.808**	**0.722**	**0.298**
TCGA-READ	SVM	**0.808**	**0.722**	**0.298**
TCGA-READ	RandomForest	**0.808**	**0.722**	**0.298**
TCGA-READ	KNN	**0.808**	**0.722**	**0.298**
TCGA-READ	RidgeClassifier	**0.808**	**0.722**	**0.298**
TCGA-READ	NaiveBayes	**0.808**	**0.722**	**0.298**
TCGA-LUSC	LogisticRegression	0.591	0.511	0.438
TCGA-LUSC	SVM	0.409	0.298	0.210
TCGA-LUSC	RandomForest	0.591	0.492	0.421
TCGA-LUSC	KNN	0.455	0.328	0.232
TCGA-LUSC	RidgeClassifier	**0.636**	**0.597**	**0.546**
TCGA-LUSC	NaiveBayes	0.409	0.323	0.257
TCGA-KIRP	LogisticRegression	0.622	0.553	0.444
TCGA-KIRP	SVM	0.511	0.407	0.268
TCGA-KIRP	RandomForest	0.578	0.482	0.351
TCGA-KIRP	KNN	0.467	0.306	0.164
TCGA-KIRP	RidgeClassifier	**0.689**	**0.659**	**0.575**
TCGA-KIRP	NaiveBayes	0.444	0.301	0.161
TCGA-KIRC	LogisticRegression	0.547	**0.511**	**0.397**
TCGA-KIRC	SVM	0.427	0.320	0.220
TCGA-KIRC	RandomForest	**0.560**	0.486	0.362
TCGA-KIRC	KNN	0.493	0.409	0.289
TCGA-KIRC	RidgeClassifier	0.520	0.490	0.374
TCGA-KIRC	NaiveBayes	0.347	0.312	0.217
TCGA-KICH	LogisticRegression	**0.750**	**0.643**	**0.429**
TCGA-KICH	SVM	**0.750**	**0.643**	**0.429**
TCGA-KICH	RandomForest	**0.750**	**0.643**	**0.429**
TCGA-KICH	KNN	0.700	0.618	0.412
TCGA-KICH	RidgeClassifier	**0.750**	**0.643**	**0.429**
TCGA-KICH	NaiveBayes	**0.750**	**0.643**	**0.429**
TCGA-ESCA	LogisticRegression	0.958	0.958	0.486
TCGA-ESCA	SVM	0.958	0.948	0.642
TCGA-ESCA	RandomForest	**0.979**	**0.969**	**0.658**
TCGA-ESCA	KNN	0.958	0.948	0.642
TCGA-ESCA	RidgeClassifier	0.958	0.958	0.486
TCGA-ESCA	NaiveBayes	0.688	0.657	0.424

Continued on next page

Table S2: Classical machine-learning performance across 17 TCGA datasets using integrated multi-omics features (dataset-wise best values in bold).

Dataset	Machine Learning Method	Accuracy	F1-weighted	F1-macro
TCGA-BLCA	LogisticRegression	0.641	0.642	**0.612**
TCGA-BLCA	SVM	0.615	0.589	0.466
TCGA-BLCA	RandomForest	0.641	0.620	0.562
TCGA-BLCA	KNN	0.436	0.406	0.406
TCGA-BLCA	RidgeClassifier	**0.667**	**0.669**	0.600
TCGA-BLCA	NaiveBayes	0.487	0.497	0.494
TCGA-ACC	LogisticRegression	0.625	0.637	0.615
TCGA-ACC	SVM	0.375	0.263	0.291
TCGA-ACC	RandomForest	0.500	0.473	0.474
TCGA-ACC	KNN	0.208	0.089	0.119
TCGA-ACC	RidgeClassifier	**0.708**	**0.720**	**0.702**
TCGA-ACC	NaiveBayes	0.417	0.358	0.369
TCGA-UCEC	LogisticRegression	**0.704**	**0.683**	**0.541**
TCGA-UCEC	SVM	0.608	0.559	0.431
TCGA-UCEC	RandomForest	0.616	0.565	0.431
TCGA-UCEC	KNN	0.424	0.398	0.310
TCGA-UCEC	RidgeClassifier	0.672	0.653	0.508
TCGA-UCEC	NaiveBayes	0.480	0.436	0.325
TCGA-STAD	LogisticRegression	**0.816**	**0.808**	**0.641**
TCGA-STAD	SVM	0.767	0.733	0.524
TCGA-STAD	RandomForest	0.806	0.787	0.621
TCGA-STAD	KNN	0.573	0.614	0.490
TCGA-STAD	RidgeClassifier	0.796	0.787	0.617
TCGA-STAD	NaiveBayes	0.583	0.538	0.289
TCGA-PCPG	LogisticRegression	**0.923**	**0.916**	**0.859**
TCGA-PCPG	SVM	0.788	0.706	0.436
TCGA-PCPG	RandomForest	0.865	0.826	0.724
TCGA-PCPG	KNN	0.769	0.717	0.514
TCGA-PCPG	RidgeClassifier	0.904	0.899	0.848
TCGA-PCPG	NaiveBayes	0.462	0.412	0.255
TCGA-LIHC	LogisticRegression	**0.895**	**0.897**	0.891
TCGA-LIHC	SVM	**0.895**	0.895	0.895
TCGA-LIHC	RandomForest	**0.895**	0.895	**0.898**
TCGA-LIHC	KNN	0.667	0.674	0.662
TCGA-LIHC	RidgeClassifier	0.807	0.808	0.801
TCGA-LIHC	NaiveBayes	0.404	0.392	0.381
TCGA-HNSC	LogisticRegression	**0.786**	**0.782**	**0.766**
TCGA-HNSC	SVM	0.607	0.593	0.565
TCGA-HNSC	RandomForest	0.726	0.720	0.705
TCGA-HNSC	KNN	0.488	0.434	0.392
TCGA-HNSC	RidgeClassifier	0.762	0.756	0.731
TCGA-HNSC	NaiveBayes	0.357	0.369	0.350
TCGA-COAD	LogisticRegression	0.859	0.839	0.561
TCGA-COAD	SVM	0.782	0.711	0.373
TCGA-COAD	RandomForest	0.833	0.770	0.451
TCGA-COAD	KNN	0.744	0.709	0.406
TCGA-COAD	RidgeClassifier	**0.872**	**0.852**	**0.575**
TCGA-COAD	NaiveBayes	0.731	0.650	0.295

Continued on next page

Table S2: Classical machine-learning performance across 17 TCGA datasets using integrated multi-omics features (dataset-wise best values in bold).

Dataset	Machine Learning Method	Accuracy	F1-weighted	F1-macro
TCGA-BRCA	LogisticRegression	**0.830**	**0.831**	**0.747**
TCGA-BRCA	SVM	0.773	0.704	0.395
TCGA-BRCA	RandomForest	**0.830**	0.797	0.587
TCGA-BRCA	KNN	0.646	0.546	0.344
TCGA-BRCA	RidgeClassifier	0.755	0.771	0.699
TCGA-BRCA	NaiveBayes	0.550	0.569	0.410

Table S3: Performance comparison on the TCGA-BRCA dataset under different random seeds. This experiment was conducted to assess the stability of the evaluation protocol.

Dataset	Omics Combination	Model	Seed	Accuracy	F1-weighted	F1-macro
TCGA-BRCA	1	simple_mlp	68	0.817	**0.812**	**0.680**
TCGA-BRCA	1	simple_mlp	2026	**0.821**	0.805	0.659
TCGA-BRCA	2	simple_mlp	68	0.803	0.799	0.665
TCGA-BRCA	2	simple_mlp	2026	**0.821**	**0.820**	**0.702**
TCGA-BRCA	3	simple_mlp	68	**0.755**	**0.752**	**0.579**
TCGA-BRCA	3	simple_mlp	2026	0.734	0.732	0.542
TCGA-BRCA	1+2	simple_mlp	68	0.817	0.814	0.694
TCGA-BRCA	1+2	simple_mlp	2026	**0.839**	**0.833**	**0.746**
TCGA-BRCA	1+3	simple_mlp	68	0.786	0.788	0.588
TCGA-BRCA	1+3	simple_mlp	2026	**0.795**	**0.793**	**0.637**
TCGA-BRCA	2+3	simple_mlp	68	0.834	0.834	0.708
TCGA-BRCA	2+3	simple_mlp	2026	**0.852**	**0.848**	**0.750**
TCGA-BRCA	1+2+3	simple_mlp	68	0.830	**0.830**	**0.735**
TCGA-BRCA	1+2+3	simple_mlp	2026	**0.834**	0.829	0.727
